# Does cone-beam computed tomography examination increase the micronuclei frequency in the oral mucosa exfoliated cells? A systematic review and meta-analysis

**DOI:** 10.1186/s12903-023-02832-3

**Published:** 2023-02-25

**Authors:** Pan Yang, Bin Xuan, Gang Li, Senrong Qi

**Affiliations:** 1 Department of Oral & Maxillofacial Radiology, Beijing Stomatology Hospital, School of Stomatology, Capital Medical University, Tian Tan Xi Li No.4, Beijing, 100050 China; 2 Department of Stomatology, Aerospace Center Hospital, Beijing, China; 3 Department of Oral & Maxillofacial Radiology, Peking University School & Hospital of Stomatology, Beijing, China

**Keywords:** Cone beam computed tomography, Micronucleus, DNA damage, Mouth mucosa

## Abstract

**Objective:**

This systematic review (SR) with meta-analysis aimed to evaluate the frequency of micronuclei in the oral mucosa exfoliated cells after cone-beam computed tomography (CBCT) examination.

**Methods:**

We performed language-independent computer-assisted data searches using PubMed databases, Cochrane, Embase, Web of Science all databases, and Google Scholar. The literature on micronucleus (MN) frequency of clinical trials before and after CBCT examination was included. The frequency of MN in exfoliated cells of the human oral mucosa was the primary outcome of the study. All statistical analyses were performed with R (version 4.1.0), RStudio (version 2022.02.2 + 485) software, and Meta packages (version 5.2–0). Two reviewers independently assessed the quality of the included studies by the EPHPP (Effective Public Health Practice Project) Modified scale with minor modifications. The heterogeneity of the data was analyzed using *I*^2^ statistics, in which* I*^2^ > 50% was considered substantial heterogeneity.

**Results:**

A total of 559 articles were selected through the search strategy. After screening titles and abstracts, nine full-text manuscripts were assessed for eligibility, and six observational studies were included in the meta-analysis. The present study showed a significant increase in MN frequency of human oral mucosal exfoliated cells 10 days after CBCT examination compared to baseline (SMD = − 0.56, 95%-CI = − 0.99 ~ − 0.13, *p* = 0.01). Because of the high heterogeneity among the studies (*I*^2^ = 72%), after removing one study that was the main source of heterogeneity, excluding the study (*I*^2^ = 47%), the common-effect model was chosen, and the meta-analysis also showed that the frequency of MN in human oral mucosa exfoliated cells increased significantly 10 days after CBCT examination (SMD =  − 0.35, 95%-CI =  − 0.59 ~  − 0.11, *p* = 0.004).

**Conclusion:**

This review suggested that CBCT examination increases the frequency of micronuclei in oral mucosal exfoliated cells.

**Supplementary Information:**

The online version contains supplementary material available at 10.1186/s12903-023-02832-3.

## Introduction

A Dental X-ray examination is an important auxiliary part of oral clinical examination. Dental X-ray examination is divided into two-dimensional plain film imaging and three-dimensional imaging techniques, including cone-beam computed tomography (CBCT) examination. Due to its high spatial resolution, CBCT has been widely used in various oral-related fields, such as diagnosing tumors, cysts and osteomyelitis in the maxillofacial region, temporomandibular arthropathy, and dental trauma, especially vertical root fracture. It is also of great help in preoperative guidance of dental implant surgery, orthodontic treatment, and endodontic treatment of complex root canals [1–3].

Although the effective dose of dental X-rays is a small percentage of the annual per capita or collective effective dose, the total number of examinations is huge. It has been increasing in recent years [4]. The dose of CBCT is higher than that of traditional oral X-ray examinations but generally lower than that of CT examinations, such as chest and abdominal CT examinations [5–9]. However, due to the different models of CBCT machines, the various scanning fields of view (FOV) and parameter settings, the absorbed dose obtained from CBCT machines varies greatly. With the enhancement of people’s awareness of health care and radiation protection, people pay more and more attention to the radiation risk of low-dose diagnostic X-ray examinations. According to epidemiological studies, multiple exposures to dental x-rays may be associated with the risk of diseases such as thyroid cancer, meningioma, laryngeal cancer, and salivary gland cancer [10, 11]. However, there is still controversy about whether a single CBCT examination will cause human body injury. Therefore, for the CBCT examination, the concepts of “as low as diagnostically acceptable” (ALADA) [12] and “as low as diagnostically acceptable being indication-oriented and patient-specific” (ALADAIP) [13] proposed in recent years.

X-rays may lead to DNA damage to cells through direct and indirect effects. Several methods evaluate the DNA damage caused by CBCT on human cells, including the micronucleus (MN) test, γ-H2ax and 53BP1 immunofluorescence test, and 8-oxo-7,8-dihydro-2′-deoxyguanosine level analysis test and so on, among which the MN test is the most widely used. Oral mucosa exfoliated cells are the most common experimental materials due to the convenience of sampling and non-trauma to the human body.

Some studies have shown that the frequency of MN in oral mucosal exfoliated cells increased significantly after CBCT examination [14–16], but there is still a controversy in the literature [17–20]. Therefore, the present systematic literature review aims to clarify whether CBCT examination increases the MN frequency in the oral mucosa exfoliated cells.

## Methods

### Protocol and registration

This study protocol was registered in the PROSPERO database (CRD42022340189) and followed the recommendations of the PRISMA statement [21].

### Information sources and search strategy

We performed language-independent computer-assisted searches of data from their inception to 18 September 2022 using PubMed databases, Cochrane, Embase, Google Scholar and Web of Science all databases. Web of Science all databases included Web of Science Core Collection, Chinese Science Citation Database, Derwent Innovations Index, KCI-Korean Journal Database, MEDLINE and SciELO Citation Index. Keywords were identified using the combination of Medical Subject Heading (MeSH) and free text Keywords of search terms shown in Table 1.

**Table 1:** Electronic database and search strategy

Database	Search strategy (September 2022)
PubMed https://www.ncbi.nlm.nih.gov/pubmed/	((((((((((((((((((((((((((((((((Computed Tomography, Cone-Beam) OR (Cone Beam Computed Tomography)) OR (CT Scan, Cone-Beam)) OR (CT Scan, Cone Beam)) OR (CT Scans, Cone-Beam)) OR (Cone-Beam CT Scan)) OR (Cone-Beam CT Scans)) OR (Scan, Cone-Beam CT)) OR (Scans, Cone-Beam CT)) OR (Tomography, Cone-Beam Computed)) OR (Tomography, Cone Beam Computed)) OR (Tomography, Volume Computed)) OR (Computed Tomography, Volume)) OR (Volume Computed Tomography)) OR (Volumetric CT)) OR (CT, Volumetric)) OR (Volumetric Computed Tomography)) OR (Computed Tomography, Volumetric)) OR (Tomography, Volumetric Computed)) OR (CAT Scan, Cone-Beam)) OR (CAT Scan, Cone Beam)) OR (CAT Scans, Cone-Beam)) OR (Cone-Beam CAT Scan)) OR (Cone-Beam CAT Scans)) OR (Scan, Cone-Beam CAT)) OR (Scans, Cone-Beam CAT)) OR (Cone-Beam Computer-Assisted Tomography)) OR (Computer-Assisted Tomography, Cone-Beam)) OR (Cone Beam Computer Assisted Tomography)) OR (Tomography, Cone-Beam Computer-Assisted)) AND (((((((((((DNA Damages) OR (Damage, DNA)) OR (Damages, DNA)) OR (DNA Injury)) OR (DNA Injuries)) OR (Injuries, DNA)) OR (Injury, DNA)) OR (Genotoxic Stress)) OR (Genotoxic Stresses)) OR (Stresses, Genotoxic)) OR (Stress, Genotoxic))) AND (((((((((Mouth) OR (Oral Cavity)) OR (Cavity, Oral)) OR (Cavitas Oris)) OR (Vestibule of the Mouth)) OR (Vestibule Oris)) OR (Oral Cavity Proper)) OR (Mouth Cavity Proper)) OR (Cavitas oris propria))
Cochrane https://www.cochranelibrary.com/library	
Embase http://www.embase.com	
Web of science all databases https://www.webofscience.com/wos/alldb/basic-search	
Google Scholar https://scholar.google.com.br/	(Computed Tomography, Cone-Beam) AND (genotoxic)

All obtained citations were exported into the endnote, where duplicates were removed.

### Eligibility criteria

The literature on clinical trials of MN frequency before and after CBCT examination was included. The frequency of MN in exfoliated cells of the human oral mucosa was the primary outcome of the study. Studies with the mean and standard deviation (SD) frequency of MN were included. If the mean and SD frequency of MN were not described in detail in the article, we contacted the corresponding author for data as possible. The exclusion criteria were as follows: (1) animal experiments and experiments were performed in vitro; (2) case reports, technical reports, abstracts for conferences, reviews, meta-analyses, or non-English literature; (3) lack of original data or unable to get a full text.

### Study selection and data extraction

All the studies were screened by two reviewers (Pan Yang and Bin Xuan). If there were any discrepancies, they would be adjudicated by the third reviewer (Gang Li). The title and abstract were read first, and the article would be read in full text if the eligibility criteria were met. After a careful full-text review, the papers that meet the eligibility criteria were included in the system review.

The following information was extracted from included studies by two researchers (Pan Yang and Bin Xuan) independently: the first author, publication year, country, number of subjects, sex, age, sampling site, sampling time, CBCT machine, CBCT scanning parameters, staining method, the total number of cells. The mean and SD in the frequency of the MN cell were obtained as primary outcomes. If there is a difference in the extracted data, it will be decided jointly through discussion with the third investigator Gang Li.

### Risk of bias in individual studies

Two reviewers (Pan Yang and Bin Xuan) independently assessed the quality of the included studies by the EPHPP (Effective Public Health Practice Project) Modified scale with minor modifications [22]. The indicators of quality assessment included the following aspects: (1) study design (number of subjects, gender distribution, and inclusion and exclusion criteria), (2) identification of confounding factors (stain and number of cells evaluated), (3) blind analysis (hide sample information when viewing cells under a microscope), and (4) data analysis (Appropriate statistical methods and analysis of cytotoxicity). According to the above four indicators, the literature was evaluated into three grades: strong (three strong ratings and no weak), weak (with two or more weak ratings), and moderate (all remaining cases).

### Statistical analysis

All statistical analyses were performed with R (version 4.1.0), RStudio (version 2022.02.2 + 485) software and Meta packages (version 5.2–0). All outcomes were continuous measures with large differences in mean values. Standardized mean differences (SMD) with 95% confidence intervals (95% CI) were used to assess changes in MN frequency before and after CBCT examinations. The heterogeneity of the data was analyzed using *I*^2^ statistics, in which *I*^2^ > 50% was considered substantial heterogeneity [23]. A random-effects model was employed if there was statistical heterogeneity (*I*^2^ > 50%). Otherwise, a common-effect model was applied. In sensitivity analysis, meta-analysis was performed after removing each study individually to evaluate the quality and consistency of the result. A *p* ≤ 0.05 was considered statistically significant in all procedures.

## Results

### Study selection

**Fig. 1:**
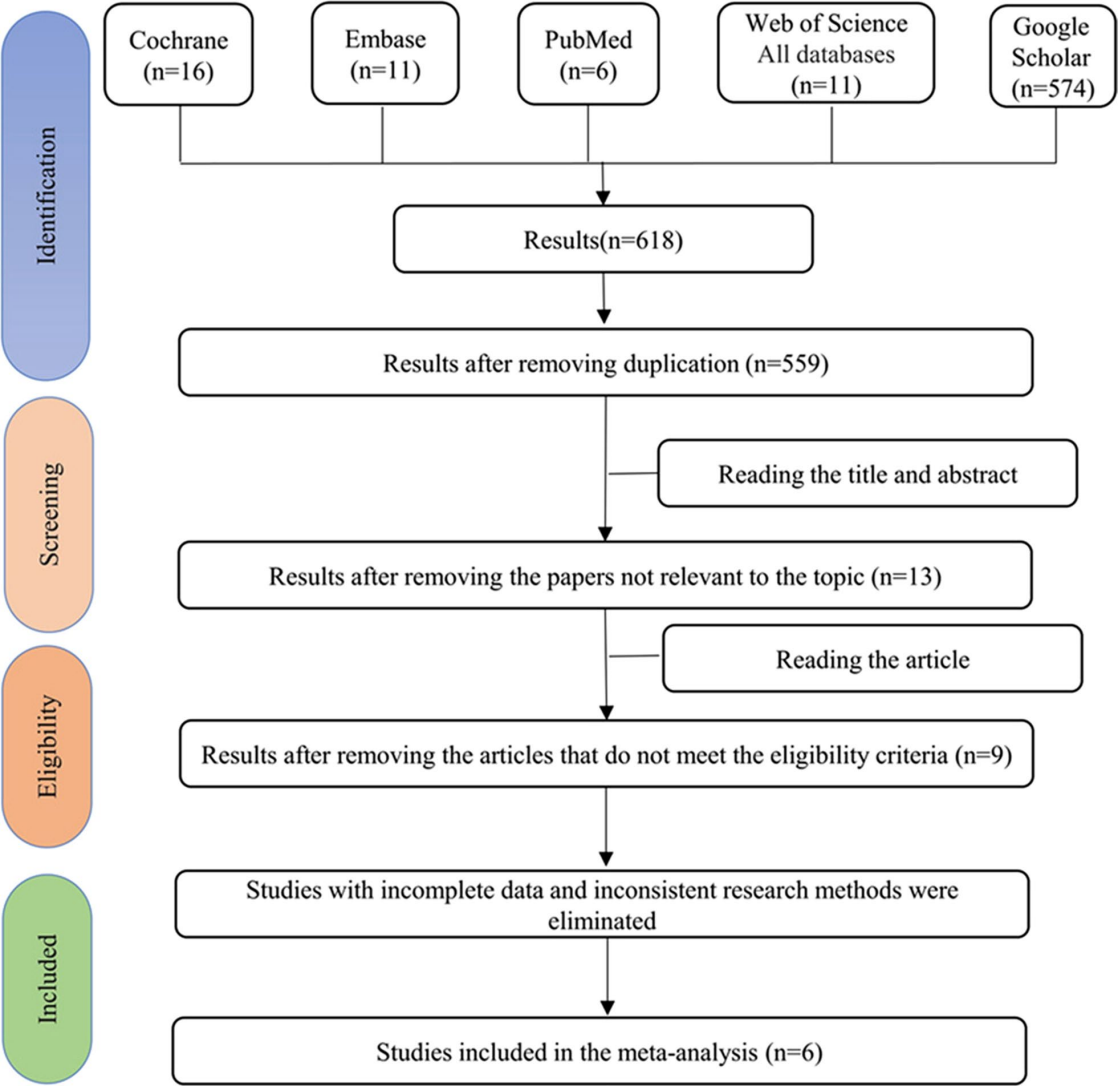
Flowchart of systematic search and study selection strategy

Figure 1 shows the literature search results and the stepwise exclusion process. The electronic database search identified 618 scientific records among which 59 were duplicates and the remaining 559 articles were screened by title and abstract. Five hundred and forty-six studies were excluded because they were irrelevant to the topic. A general review of articles, an in vitro study, a technical report and an abstract for conferences were excluded. We carefully read the remaining nine full-text articles, 3 of which were excluded due to incomplete data and inconsistent research techniques. There was only the mean of MN and no SD value in the study of Li et al. [8]. In da Fonte JB's study [17], accurate mean and SD values were not obtained because the results were represented by the statistical graphs. In addition, Mounika’s [24] study was divided into the single CBCT exposure group and the double CBCT exposure group, and the sampling time was inconsistent with other studies. Although the mean and SD values for the number of micronuclei were explained, this study was excluded because we can’t calculate the frequency since we don’t know how many exfoliated cells were counted. Finally, six works of literature could be used for meta-analysis.

### Study characteristics

A summary of the studies included in the present systematic review was shown in Table 2. Among the six studies, two studies were conducted in Brazil and other studies were conducted in China, Egypt, Saudi Arabia, and Iran. Six studies involved 167 subjects, ranging from 9 to 50 years. Five studies included males and females, and one had only males.

**Table 2:** Characteristics of the studies selected for this systematic review

Study ID	Country	Exclusion criteria	Number of subjects	Age	Sex	Site of sample	Sampling time	CBCT machine	Parameter settings
Carlin [15]	Brazil	Yes	19	26.8 ± 5	10 males and 9 females	buccal mucosa	before CBCT exposure and after 10 days	i-CAT	80 kV, 4 mA, 40 s
Lorenzoni [14]	Brazil	Yes	24	11 ± 1.2	14 males and 10 females	buccal mucosa	before CBCT exposure and after 10 days	i-CAT	FOV: 13 × 13 cm (120 kV, 46.72 mAs, 40 s) and FOV: 22 × 22 cm (120 kV, 47.74 mAs, 40 s)
Yang [16]	China	Yes	46	23–42	7 males and 39 females	buccal mucosa, tongue, keratinized mucosa	before CBCT exposure and after 10 days	NewTom VG	FOV: 15 × 15 cm, 110 kV,6.24–14.45 mA, dose of area product (DAP) 448.15–730.79 mGy cm^2^
Basha [19]	Egypt	Yes	30	27–43	30 males	buccal mucosa	before CBCT exposure and after 10 days	J Morita	FOV: 8 × 10 cm, 84 kV, 9–14 mA, 6 s, 0.16 mm voxels
Altoukhi [18]	Saudi Arabia	Yes	18	9–12	8 males and 10 females	buccal mucosa	before CBCT exposure, after 10 ± 2 days, and after 1 month	i-CAT	FOV: 16 × 6 cm, 120 kV, 10 mA, 4.8 s and 0.4 voxels. The total effective dose was around 22µSv
Mosavat [20]	Iran	Yes	30	20–50	5 males and 15 females	buccal mucosa	before CBCT exposure and after 10 days	3030 Alphard VEGA	FOV: 10 × 10 cm, 80 kV, 4 mA, 17 s

Stain methods	Number of cells evaluated	Blind analysis description	Consistency check	Mean of MN before CBCT	Sd of MN before CBCT	Mean of MN 10 days after CBCT	Sd of MN 10 days after CBCT	*P*-value	Other cytogenetic
Feulgen/Fast Green	2000	No	No	0.04	0.05	0.05	0.06	*P* > 0.05	other nuclear alterations (karyorrhexis, pyknosis and karyolysis)
Feulgen/Fast Green	1000	Yes	Yes	0.025	0.07	0.033	0.08	*P* > 0.05	other nuclear alterations (karyorrhexis, Pyknosis and Karyolysis)
Feulgen/Fast Green	1000	Yes	Yes	0.37	0.572	0.46	0.721	*P* > 0.05	nuclear buds, basal cells, binucleated, condensed chromatin, pyknosis, karyolysis, karyorrhexis
Papanicolaou stain	1000	Yes	No	0.026	0.0062	0.03	0.0068	*p* < 0.001	other nuclear changes (pyknosis, condensed chromatin and karyorrhexis)
Feulgen/Fast Green	1000	Yes	Yes	0.3	0.3	2.2	2.3	*p* = 0.00	Condensed chromatin Karyorrhexis Pyknosis Karyolysis
Papanicolaou stain	1000	Yes	Yes	5.13	1.73	7.67	2	*p* < 0.0005	Pyknosis, Karyorrhexis, karyolysis, budding, binucleation, cytotoxicity

Different CBCT machines were used in the studies. I-CAT CBCT (Imaging Sciences International, Hatfield, Pa) was used in three of the studies, and the others were NewTom VG (Quantitative Radiology, Verona, Italy), J Morita (J. Morita, Corporation, Kyoto, Japan), and 3030 Alphard VEGA (Asahi, Roentgen. Ind. Co. Ltd., Kyoto, Japan) respectively. However, the Settings of machine parameters for CBCT examination varied widely. The FOV ranged from 8 × 10 cm to 22 × 22 cm, the tube voltage ranged from 80 to 120 kV, the tube current ran from 4 to 14 mA and the exposure time went from 4.8 s to 40 s.

All the included studies showed that the subjects had not received CBCT or other X-ray irradiation in the previous 16 days to 6 months. Samples were taken before CBCT exposure and 10 days after in all six studies, and additional analysis was conducted 1 month after CBCT scanning in Altoukhi’s study [18]. All studies smeared the mucosa of the buccal. The cells of the tongue and gingival cells were also collected in the study of Yang et al. [16]. The number of cells counted per participant was usually 1000, while Carlin’s study performed 2000 cells counting per participant. The Feulgen and fast green stain technique was the most commonly used staining method, applied in four studies, two studies used the Papanicolaou staining technique.

All studies not only evaluated MN cells but also evaluated other cytogenetic alterations. Two studies evaluated other nuclear alterations (karyorrhexis, pyknosis and karyolysis), while one study evaluated other nuclear changes (pyknosis, condensed chromatin and karyorrhexis). The remaining three studies assessed different types of cellular changes separately, including nuclear buds (two studies), basal cells, binucleated cells (two studies), condensed chromatin (three studies), pyknosis (three studies), karyolysis (three studies), karyorrhexis (three studies) and cytotoxicity.

### Main findings

The main findings of these studies were shown in Table 3. As we suspected, these experimental results are contradictory regarding whether CBCT can cause genetic damage to cells. Basha [19], Altoukhi [18], and Mosavat [20] proved that the frequency of MN in oral exfoliated cells increased significantly 10 days after exposure to CBCT. However, Carlin [15], Lorenzoni [14], and Yang [16] did not find a statistical difference increase in MN frequency after exposure. According to the study of Basha et al. [19], there was no statistically significant difference in the mean percentage of MN between one month later and before irradiation.

**Table 3:** Main findings of the studies selected for this systematic review

Study ID	Main findings	
	Cytotoxicity	Genotoxicity
Carlin [15]	Other nuclear alterations (karyorrhexis, pyknosis and karyolysis) ↑	MN no significant differences
Lorenzoni [14]	Other nuclear alterations (karyorrhexis, pyknosis and karyolysis) ↑	MN no significant differences
Yang [16]	Pyknosis ↑ Karyolysis↑	MN no significant differences
Basha [19]	Other nuclear changes (pyknosis, condensed chromatin and karyorrhexis)↑	MN↑
Altoukhi [18]	Condensed chromatin↑ Karyorrhexis ↑ Pyknosis ↑ Karyolysis↑	MN↑
Mosavat [20]	Pyknosis ↑ Karyorrhexis ↑ Karyolysis ↑ Budding ↑ Cytotoxicity*↑	MN↑ p53 expression↑

*Cytotoxicity was determined as the sum of all cases of pyknosis, karyorrhexis, karyolysis, budding and binucleation

Regarding cytotoxicity, all studies have found different types of cytotoxic increases in oral mucosa cells of patients submitted to CBCT after 10 days of exposure. The relevant results of these studies were shown in Table 3. In addition, according to the study of Basha et al. [19], compared with the basic level, one month after CBCT examination, there was a significant increase in karyolysis, and there was no significant difference in the condensed chromatin, pyknosis, and karyorrhexis.

According to the research of Altoukhi [18], gender is not a significant factor affecting the mean percentage of MN, condensed chromatin, karyorrhexis, pyknosis, or karyolysis. According to the study of Basha [19], there was no significant difference between the increase of MN cells and the age of patients.

### Assessment of the risk of bias

The quality assessment of selected studies is shown in Table 4. EPHPP standards evaluated the quality of different indicators, four studies were classified as moderate [14, 15, 19, 20] and two studies as strong [16, 18]. Therefore, the quality of all included studies can reach a good indicator for evaluating MN after CBCT examination.

**Table 4:** Quality assessment and a final rating of the studies

Author	Study design	Confounders	Blinding	Data analysis	Final rating
Carlin V	Moderate	Strong	Moderate	Moderate	Moderate
Lorenzoni DC	Moderate	Moderate	Strong	Moderate	Moderate
Yang P	Strong	Moderate	Strong	Strong	Strong
Basha S	Moderate	Weak	Moderate	Moderate	Moderate
Altoukhi DH	Strong	Moderate	Strong	Strong	Strong
Mosavat F	Strong	Weak	Strong	Strong	Moderate

### Meta-analysis

**Fig. 2:**
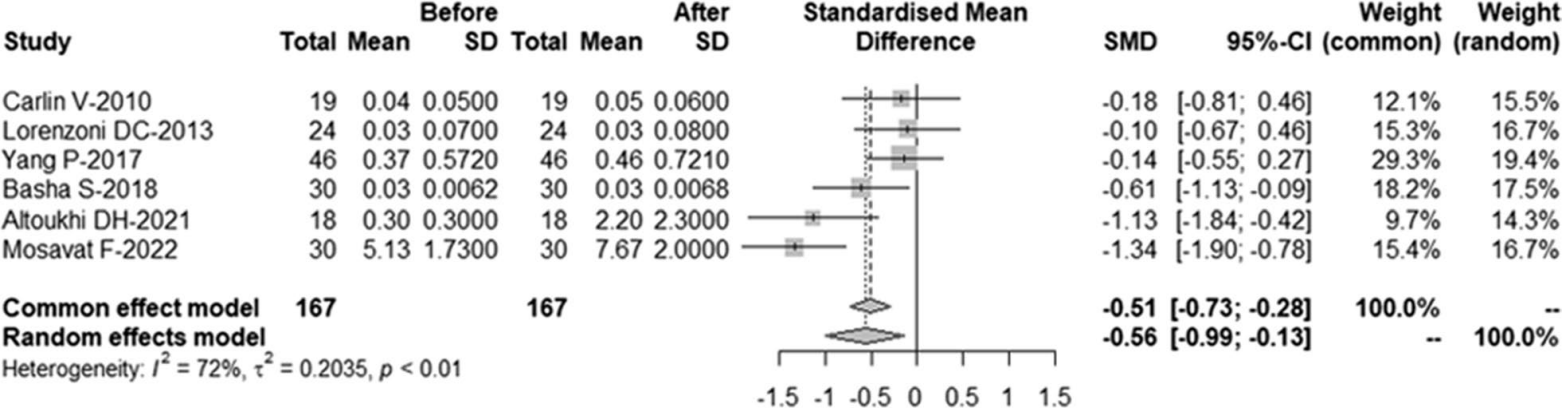
Forest plot of the frequency of micronuclei for CBCT examination before vs 10 days after

Due to the high heterogeneity among the studies (*I*^2^ = 72%), a random effects model was used, the meta-analysis showed that MN frequency of human oral mucosal exfoliated cells increased significantly in 10 days after CBCT examination compared to baseline (SMD = − 0.56, 95%-CI = − 0.99 ~ − 0.13, *p* = 0.01). The result was shown in Fig. 2.

**Fig. 3:**
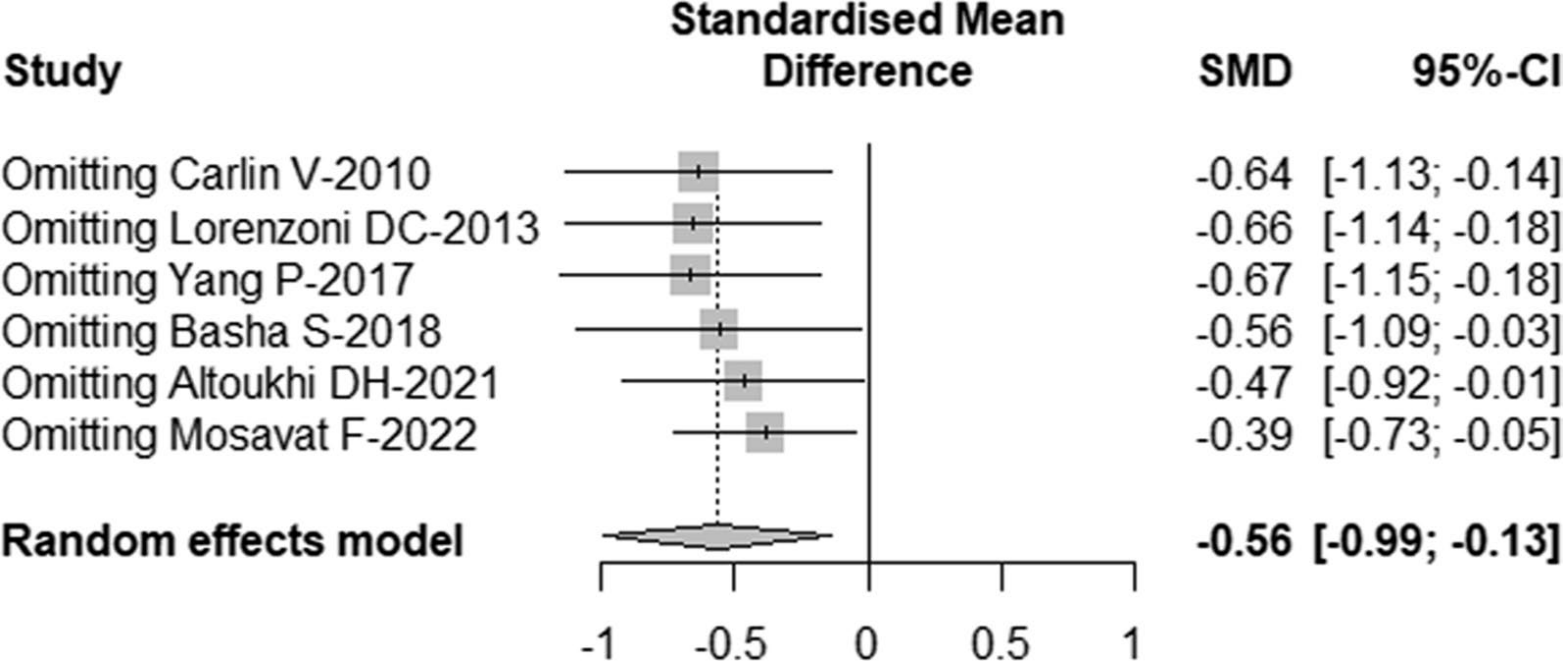
Forest plot of sensitivity analysis

**Fig. 4:**
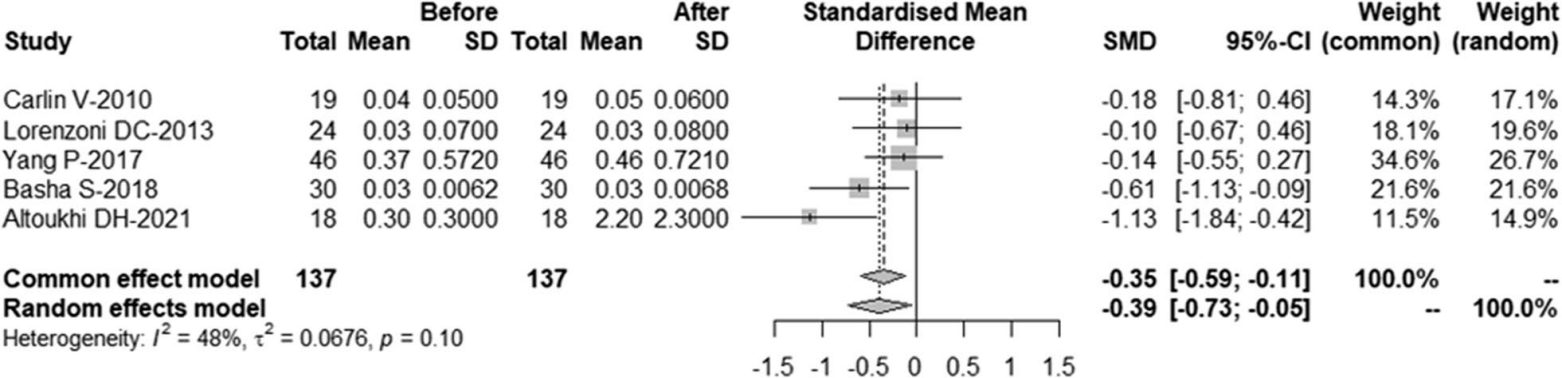
Forest plot of the frequency of micronuclei after removing the study with high heterogeneity

Removing each study individually, the results revealed that one study by Mosavat et al.[20] was the main source of heterogeneity (Table 5, Fig. 3). After excluding the study (*I*^2^ = 47%), the common-effect model was chosen and the meta-analysis also showed that the frequency of MN in human oral mucosa exfoliated cells increased significantly 10 days after CBCT examination (SMD = − 0.35, 95%-CI = − 0.59 ~ − 0.11, *p* = 0.004). The result was shown in Fig. 4.

**Table 5:** Sensitivity analysis was conducted by omitting each study individually and re-analyzing the remaining studies

	SMD	95%-CI	*I*^2^
Omitting Carlin-2010	− 0.6375	[− 1.1307; − 0.1442]	76%
Omitting Lorenzoni-2013	− 0.6566	[− 1.1375; − 0.1756]	74%
Omitting Yang-2017	− 0.6661	[− 1.1507; − 0.1816]	70%
Omitting Basha-2018	− 0.5595	[− 1.0894; − 0.0296]	77%
Omitting Altoukhi-2021	− 0.4684	[− 0.9235; − 0.0134]	72%
Omitting Mosavat-2022	− 0.3878	[− 0.7254; − 0.0501]	48%

## Discussion

Ionizing radiation directly acts on the DNA, proteins and other macromolecules of the organism, or generates free radicals to cause further a series of biological effects, including DNA damage and chromosomal mutation, which may be the cause of cancer. The MN assay is the most commonly used genotoxicity test to detect chromosomal damage. MN is considered because after physical or chemical stimulations, the chromosome fragments that lost the centromere at the anaphase of mitosis cannot enter the main nucleus at the end of the division, and form a small nucleus outside the main nucleus, namely MN. Oral mucosal tissue is in constant renewal, and the renewal speed is fast and the cycle is short, usually 7–14 days [25]. Therefore, the changes in oral mucosal exfoliated cells on the 10th day after X-ray irradiation can reflect the radiation-induced cytological and cytogenetic changes. According to the present meta-analysis, the results showed that the frequency of MN increased significantly 10 days after exposure to CBCT examination, which supported the preliminary hypothesis that the radiation dose of a single CBCT scan may cause chromosomal aberrations in oral mucosal exfoliated cells. This result is consistent with the results of a previous meta-analysis of Santos [26], which proved that panoramic X-ray film could also produce a small extent of genotoxic damage to oral mucosa cells. Since the dose of CBCT was significantly higher than that of panoramic radiation, this result was logical and expected.

Many factors during the experiment can affect the experimental results. The different sampling sites of oral mucosal exfoliated cells may also be one of the reasons that affect the results. The bilateral buccal mucosa is the most common sampling site; however, the gingiva and dorsum of the tongue are occasionally sampled. Several biomarkers of genetic damage and cell death were observed in both lymphocytes and buccal cells, so buccal mucosal cells are recommended [25]. In addition, according to the experimental results of Yang et al. [16], there was no statistical significance in the changes of MN and other types of cytotoxic in the exfoliated cells of buccal, tongue and gingival mucosa, which was consistent with the experimental results of Arora [27] on buccal and gingival mucosa.

There are many staining methods for MN, including non-DNA specific staining and DNA-specific staining such as Feulgen or Acridine orange [28]. Feulgen stain was recommended because stained DNA can be specifically observed under a fluorescence wavelength of 580–620 nm to avoid confusion of other particles [29]. Different staining methods have a great influence on the frequency of MN. If non-specific staining methods are used, false positive results may occur. As the frequency of MN in the exfoliated cells of oral mucosa is low both before and after CBCT irradiation, the larger the count is, the more accurate the results. At least 2000 normally differentiated cells would need to be scored at ×1000 magnification to identify DNA damage biomarkers as suggested by Thomas P [29]. In this systematic review, only 1 study counted 2000 cells, while all others counted 1000 cells per volunteer. To compensate for the different coloring methods and the fact that the mean of the results is very different, standardized mean difference instead of mean difference was used to calculate the study results.

The inclusion of subjects may also affect the results. Smoking, alcohol use, drug use, and systemic diseases may also affect the frequency of MN cells. In the literature included in the present system evaluation, there were inclusion and exclusion criteria for subjects to reduce other factors’ interference in the results. According to current studies [18, 27, 30], gender has no effect on the MN rate after an oral X-ray examination. However, whether age has an impact on the MN rate is still controversial. According to the study by Basha [19], the increase of MN cells was not significantly related to the age of patients. This result is consistent with Li's study [8], which shows that no statistically significant differences were observed between adult and juvenile subjects, regardless of genotoxicity or cytotoxicity. However, according to the results of Popova [30] and Arora [27], MN cell rate was correlated with the age of subjects undergoing panoramic examinations.

In addition to MN cell changes, other types of cell changes have also attracted our attention. Because when cells are exposed to ionizing radiation, it is possible to eliminate genetically damaged cells through the process of apoptosis. Therefore, the increase in apoptosis is also an important indicator of cell damage [31]. Pyknosis, condensed chromatin and karyorrhexis were probably the early stages of apoptosis, and with the process of cell death, the nucleus is completely dissolved in the late stage [29]. Therefore, pyknosis, condensed chromatin, karyorrhexis and karyolysis were all representatives of cytotoxicity. Regarding cytotoxicity, all studies have found different types of cytotoxic increases in oral mucosa cells of patients submitted to CBCT after 10 days of exposure. However, some of the studies described each type of cytotoxicity cell separately, and some of the studies analyzed the sum, so it was impossible to unify these results for meta-analysis.

The lack of measurements of radiation dose absorbed by patients in the included studies is one of the limitations of this systematic review. Due to the different machines of CBCT, as well as the variation of the field of view and irradiation parameters setting, the absorbed dose of patients with CBCT varies greatly. According to the meta-analysis of CBCT dose by Ludlow et al. [32], the results showed that the average effective dose of large-field irradiation for adults was 212 μSv, 177 μSv for middle-field irradiation and 84 μSv for small-field irradiation. The mean effective dose was 175 μSv for large/medium fields and 103 μSv for small fields for children. Unfortunately, only one study in the included literature described the absorbed dose of subjects. Other literature did not provide information on the absorbed dose, so further analysis of radiation dose and injury was impossible. In addition, we did not perform publication bias tests because the number of included literature was less than ten [33].

## Conclusion

Despite the limitations above, the present study demonstrated that CBCT examination increased the micronuclei frequency of exfoliated cells in the oral mucosa. Although CBCT is very helpful for diagnosing and treating oral diseases, doctors should be alert to the potential risks that this test may pose. Therefore, CBCT should only be prescribed by a physician when conventional radiographs do not serve the purpose. However, there is no need to panic among the public, the damage caused by CBCT examinations is still at a low level and most of the damage may be repaired or cleared over time. However, we still need to strictly follow the three principles of radiological protection: the justification of practice, the optimization of radiation protection and the limitation of individual dose.

## Supplementary Information

**Additional file 1**. Raw data of micronuclei frequency before and after CBCT examination in the included studies.

**Additional file 2**. PRISMA checklist for this systematic review.

## Data Availability

The datasets supporting the conclusions of this article are included in Additional files 1 and 2.

## Abbreviations

SR: Systematic review
CBCT: Cone-beam computed tomography
FOV: Fields of view
MN: Micronucleus
SD: Standard deviation
SMD: Standardized mean differences
