# Supplementary material for: Does cone-beam computed tomography examination increase the micronuclei frequency in the oral mucosa exfoliated cells? A systematic review and meta-analysis
Source: BMC Oral Health. 2023 Feb 25;23:127. doi: 10.1186/s12903-023-02832-3 (PMC9960480; doi:10.1186/s12903-023-02832-3)
Supplement: Supplementary file 1 — Additional file 1. Raw data of micronuclei frequency before and after CBCT examination in the included studies. [file 12903_2023_2832_MOESM1_ESM.docx]

Additional file1. Raw data of micronuclei frequency before and after CBCT examination in the included studies

| Author | Year | Number of subjects | MN before CBCT | | MN after CBCT | |
| --- | --- | --- | --- | --- | --- | --- |
|  |  |  | Mean | sd | Mean | sd |
| Diego Coelho Lorenzoni | 2013 | 24 | 0.025 | 0.07 | 0.033 | 0.08 |
| V Carlin | 2010 | 19 | 0.04 | 0.05 | 0.05 | 0.06 |
| Doua H. Altoukhi | 2021 | 18 | 0.3 | 0.3 | 2.2 | 2.3 |
| Soha Basha | 2018 | 30 | 0.026 | 0.0062 | 0.03 | 0.0068 |
| Farzaneh Mosavat | 2021 | 30 | 5.13 | 1.73 | 7.67 | 2 |
| pan yang | 2017 | 46 | 0.37 | 0.572 | 0.46 | 0.721 |
